# Ethical Management of Elective Surgery Waiting Lists: A Framework of Clinicians’ Values in Action

**DOI:** 10.1007/s11673-025-10481-0

**Published:** 2025-10-13

**Authors:** Sharon Feldman, Katheryn Hall, Danielle Ko, Rosalind McDougall

**Affiliations:** 1 Department of Paediatrics, University of Melbourne, Melbourne, VIC Australia; 2 Children’s Bioethics Centre, Royal Children’s Hospital, Parkville, VIC Australia; 3 Clinical Ethics and Decision Support Unit, Department of Surgery, University of Melbourne at Austin Health, Heidelberg, VIC Australia; 4 Melbourne School of Population and Global Health, The University of Melbourne, Melbourne, VIC Australia

**Keywords:** Elective surgery, Planned surgery, Ethical framework, Ethical decision-making, Clinical ethics

## Abstract

Waiting time for elective surgery in public health systems has long been a focus for health institutions and governments. Such focus, and existing ethics literature, tends to hover at a policy or systems level. However, long waiting lists also create ethical challenges at the level of individuals’ clinical practice. This project expands the focus of the elective surgery waiting list discussion from the macro systems level to the micro clinician level. We put forward a framework of values driving clinicians’ ethical practice in this area, based on a literature review and iterative ethicist-led discussions with eighteen key surgical staff in one hospital network in metropolitan Melbourne, Australia. While sensitive to population-level values at play, clinicians’ primary moral orientation was towards the individual patients they cared for. This orientation saw clinicians implement additional values in their practice including supporting patient autonomy through informed consent, maximizing benefits to individuals by considering patients holistically, minimizing harms to individual patients related to time spent waiting, and consistency of decision-making within teams and departments. By articulating values that can be translated into action within clinicians’ sphere of agency, the framework aims to support ethical practice among surgical staff working within a constrained and imperfect system.

**Supplementary Information:**

The online version contains supplementary material available at 10.1007/s11673-025-10481-0.

## Introduction

Waiting time for elective surgery in the public health system has garnered attention for some time. Demand for elective surgery in Australia has been observed to increase over the past two decades, with the ageing population being a key factor (Royal Australian College of Surgeons 2011). As well as being of significant consequence to individual patients, it is often used as a proxy for the overall state of the health system, making it a priority of health institutions and governments. Since the COVID-19 pandemic, attention to planned surgery waiting lists has been increasingly urgent and sustained, reflective of the concern and, in some cases, distress, of various stakeholders.

In our context in Melbourne (in the state of Victoria, Australia), long periods of lockdown during the COVID-19 pandemic had a pronounced effect on planned surgery waiting lists. In the Victorian public health system, according to the planned surgery access policy (Victorian State Government, Department of Health 2024), patients awaiting surgery are classified into one of three urgency categories that take into account the likelihood of clinical deterioration and recommended time frames for surgery. Category 1 patients are considered the most urgent, with surgery clinically indicated within thirty days. For category 2 and category 3 patients, surgery is clinically indicated within ninety and 365 days respectively. A key principle of the Victorian policy is that within these urgency categories, patients should be “treated in turn.” That is, that whenever possible, within urgency categories, those who have been waiting the longest should receive their surgery next.

With 150 days of restrictions on planned surgeries during lockdowns, along with ongoing staff and other resource shortages, waiting list numbers and waiting times for surgery in the Victorian public system doubled between March 2020 and March 2022 (Drysdale et al. 2023). By the end of January 2022 there were 83, 341 patients on the list, waiting an average of 212 days, compared with 43, 073 waiting an average of 99 days in January 2019 (Drysdale et al. 2023). At the time this project was conceived, waiting lists in some clinical areas were being described in the media as “catastrophic”, with patients facing “agonising” waits for much-needed surgeries (Cunningham 2023, ¶3, ¶9). While the Victorian public health system has made significant progress working through the backlog (Victorian Agency for Health Information n.d.),^1^ demand continues to exceed supply. Notably, this has always been the case in planned surgery, where resources are not scarce but are finite, leading to ongoing management challenges in the context of ever-increasing demand for all types of surgery.

When it comes to elective surgery waiting lists, health institutions and governments focus on a policy or systems level. This is also reflected in ethics literature, which tends to focus on macro level justice questions about fair prioritization (Pugh et al. 2023; Curtis et al. 2010). However, long waiting lists also create ethical challenges at the level of individual clinical practice. Working within this strained system has a significant impact on individual clinicians and creates clinical ethics challenges. This project was initiated following clinical ethics discussions with surgical staff in which they described the challenging ethical situations that they faced. As well as concern about potential poor outcomes for individual patients, clinicians were worried about how to fulfil their duty of transparency to patients about wait times and the flow-on implications for the unit’s other patients when surgical time and theatre space was allocated to a specific patient’s procedure.

This project therefore expands the focus of the elective surgery waiting list discussion from the macro systems level to the micro clinician level, recognizing the experiences and agency of clinicians working in this area. The aims of this project were: 1) to articulate clinicians’ concerns and the values that inform their practice in relation to planned surgery waiting lists; and 2) to develop ethics resources to guide clinicians and surgical unit leadership in relation to key decisions. These aims were achieved through a ground-up, practice-based process, with an initial literature review supplemented by iterative ethicist-led conversations with key staff. This process generated a values framework, extending the existing literature to capture additional values implemented by clinicians in their everyday work in this area of practice. We have termed this overall set of values as “values in action” to emphasize their grounded nature. This set of values serves to clarify the ethical challenges in this area by highlighting what is at stake, and can inform clinicians’ practical responses. We also developed a detailed map of clinicians’ ethical challenges reported separately elsewhere.

Relating to terminology, elective and planned surgery are terms that can be used interchangeably. In our context, government has moved to using the term “planned” surgery (Victorian State Government, Department of Health, 2024). However, the term elective surgery is still used as everyday language by clinicians. For the purposes of this paper, we adopt the term planned surgery as, in our view, it better captures the nature of the procedures, which are medically necessary but unlike emergency surgeries can be planned ahead of time.

### Ethical Guidance for Clinicians in the Existing Ethics Literature

The first stage of this project was to conduct a critical interpretive literature review to determine what ethical guidance exists for clinicians and leaders working in the planned surgery space. Critical interpretive review is a method of literature review specific to the field of bioethics. Distinct from a systematic review that answers a research question with a comprehensive and pre-set approach based on methods in the biosciences*,* a critical interpretive review aims to capture and analyse key ideas relevant to an ethics question with flexibility and rigour (McDougall 2015). We were specifically seeking literature that proposed ethical frameworks, principles, or values relevant to the management of planned surgery waitlists.

Literature searches were conducted in March and September 2023. In March 2023, titles, abstracts, and keywords were searched in Scopus and PubMed to identify literature published from 2020 onwards (post COVID-19 pandemic) that proposes ethical frameworks, principles, or values relevant to the management of planned surgery waitlists. A Google Scholar search was also conducted at this time, with the first four pages of hits reviewed. In September 2023, a search strategy for MEDLINE (Ovid) was developed by an experienced informationist. This search was guided by the same research question but was not limited by date. Recent publications (not yet indexed) of specific bioethics journals and the reference list of all included publications were also screened for additional content. Based on these searches, a total of 110 papers were screened, and eleven papers were included in the review. Further details of the review process and included papers, including PRISMA diagram, are provided in a supplementary file.

In brief, while acknowledging the “urgent need” for ethical frameworks (Chew et al. 2020, 873), guidance on prioritization (Karamanou, Vrachatis, and Tousoulis 2020), and determination of key ethics questions (Curtis et al. 2010), the literature does not provide clear ethical guidance on how to manage the various ethical challenges related to planned surgery waitlists in practice. Various prioritization tools are canvassed in the literature, however they are not assessed in relation to their ethical merit (Rathnayake, Clarke, and Jayasinghe 2021). Two papers cite the four principles of bioethics (Beauchamp and Childress 2001) as a key framework of ethical analysis but note the difficulty of resolving the ethical challenges in this context with their application (Karamanou, Vrachatis, and Tousoulis 2020; Macleod, Mezher, and Hasan 2020).

What can be gleaned from the literature is a set of key ethical values relevant to planned surgery, including both substantive and procedural values. In terms of substantive values, the most cited are fairness/equity and maximizing benefits at a population level. The key procedural values that emerge are transparency, certainty, and scientific validity. These values are outlined in Table 1. Notably, the literature raises more questions than it answers as to how these values—in particular, the substantive values—should be operationalized in practice.

**Table 1:** Key values gleaned from the literature

**Substantive Values**	
Fairness/equity	Noted to be difficult to determine or define. In this literature, the terms are used interchangeably to refer to both treating individuals equally and ensuring equality of outcomes among individuals when making prioritization decisions.
Maximizing benefits at a population level	Prioritization decisions should achieve the greatest good for the population overall.
**Procedural Values**	
Transparency	Prioritization processes should be well-defined and open to public scrutiny.
Certainty	Patients ought to have clear and accurate expectations around the timing of their treatment.
Scientific validity	Decisions to offer surgery, as well as prioritization tools, should be evidence-based.

While there is apparent agreement that fairness/equity is key to ethically appropriate decision-making in this context, as Pitt et al. succinctly put it, “fairness is not easy to determine or define” (Pitt et al. 2003, 174). We note that the terms fairness and equity are sometimes considered to have distinct meanings, with fairness generally related to equality in treatment and equity to equality in outcomes (Rescher 2018). However, in this body of literature, the two terms are used interchangeably to refer to both equality in treatment and in outcomes. In this paper, we will also use these terms interchangeably. Some standard approaches to achieving fairness are a first-come, first-served approach, or a lottery system, which arguably give people an equal chance to access a scarce resource (Pugh et al. 2023). While time spent waiting is arguably morally significant, neither of these approaches alone is considered appropriate for achieving fairness in planned surgery waitlists (Pugh et al. 2023). One commonly proposed (and adopted) approach to achieving equity/fairness is first to prioritize patients according to clinical need. However, we are then left with the question of how to determine clinical need. As Pugh et al. (2023, 2) highlight, “it would be a mistake to assume that [prioritising according to clinical need] requires only a clinical or scientific judgement”—it requires ethical judgments as well. Key questions in relation to defining clinical need include whether psychosocial factors should be taken into account, and whether disadvantaged groups should be given preferential treatment (Curtis et al. 2010; Pugh et al. 2023).

Prioritizing patients according to clinical need is also one way to enact the second substantive value that emerges in the literature: maximizing benefits at the population level. This value is essentially a utilitarian one, seeking to achieve the greatest good overall in the population. Although, as Pugh et al. (2023) highlight, the “prioritarian” approach of categorizing patients according to clinical need may not always maximize overall benefits amongst a population. One reason for this is that a prioritarian approach may de-prioritize those likely to develop significant need in the future if not treated now. Additional questions arise as to how to operationalize the value of maximizing benefits at a population level, including how to define benefits and how to compare and therefore prioritize them. Using measures such as a Quality-Adjusted Life Year (QALY)—one measure used in health economics to assess the value of a medical intervention—can “raise concerns about equality, discrimination, moral status, and the proper role of medicine” (Pugh et al. 2023, 4). Assessments of benefit are also subjective and may require clinicians to make judgments that are not purely clinical (Pugh et al. 2023).

The remaining key values that can be gleaned from the literature are procedural and arguably can be operationalized more straightforwardly. While procedural values do not solve the fundamental ethical challenge of how to allocate resources, they are an important complement that “shape and constrain” the translation of substantive values into prioritization practices (Emanuel, Upshur, and Smith 2022, 1544). Transparency is highlighted in the literature as a key procedural value in relation to planned surgery; prioritization processes should be well-defined and open to public scrutiny (Curtis et al. 2010; Pitt et al. 2003). Certainty is also promoted as an important value in this context. Patients ought to have clear and accurate expectations around the timing of their treatment. Finally, scientific validity is emphasized as a crucial procedural value. This is relevant to determining potential benefits of interventions for individual patients, as well as implementing tools and systems for prioritization (Curtis et al. 2010). As Pitt et al. (2003, 175) aptly state, “[a] process that is evidence-based, transparent and accountable … will go a long way to making people feel that even if they haven’t received a high priority, at least they have been dealt with fairly.”

While the literature does highlight key values that are important to consider when managing planned surgery waitlists, many questions remain about how to operationalize these values in practice. Further, the principal focus of this literature is on the macro resource allocation challenge of planned surgery—how patients on waiting lists should be prioritized—that is generally dealt with at a policy or systems level. It does not acknowledge the micro ethical challenges faced by clinicians practising in this space nor all of the values that guide their decision-making at the individual or unit level.

## Conversations With Key Clinicians About Ethical Practice

After completing the literature review, we held a series of conversations with individual clinicians and managers working in planned surgery. The aims of these ethicist-led conversations were:

- to understand the relevance of these values to clinicians' practice,
- to identify the ethical challenges clinicians and leaders face, and
- to gain further insight into how decisions are made in practice.

These conversations were led by a clinical ethicist (SF) and a research nurse with extensive clinical ethics experience (KH). Following each conversation, we noted the challenges and values discussed, which informed future conversations in an iterative process. The broader research team met regularly to discuss the issues and values identified from the conversations, working together to articulate the ultimate values framework produced as well as the related practical clinical ethics advice that draws on both clinicians’ and ethicists’ insights. We sought feedback about the values framework and practical advice from the clinicians with whom we spoke to ensure that these outputs accurately reflected their experience.

In total, we spoke with eighteen clinicians and managers from a broad range of specialties and roles across hospitals within a hospital network in Melbourne, Australia. Represented units included the Hepatobiliary Surgery Unit, Gynaecology, Colorectal, Orthopaedics and Upper Gastrointestinal, and Endocrine Surgery Unit. We also spoke with members of the clinical operations division. Represented roles included surgical liaison nurses, surgical services management, physiotherapists, surgical fellows, consultants, heads of unit, and department directors. Ethics approval for the project was granted by the Austin Human Research Ethics Committee *(HREC/102419)*.

## Identifying Additional Values in Action

In the ethical challenges described to us by clinicians, some of the values at stake were those that we identified in our literature review, including equity, maximizing benefits at a population level, transparency, and certainty. Clinicians did not always explicitly name these values; rather, they described ethical challenges in ways that pointed to these values being at stake. Sometimes clinicians *did* specifically refer to values identified in the literature review but using different terminology. For instance, “efficiency of the system” was often raised by clinicians as an important consideration, which in context could be understood as concern for maximizing benefits and minimizing harms at a population level, by preventing delays and reducing waste within the system.

As noted above, the literature considers planned surgery management at a population or systems level, with the values that it highlights geared towards justice and beneficence at this same macro level. While also sensitive to these “macro values” at play, those we spoke with were more focused on decisions within their sphere of agency. Further, their primary moral orientation was towards the individual patients they care for and manage. This orientation saw clinicians implement other specific values in their practice including: 1) supporting patient autonomy through informed consent; 2) maximizing benefits to individual patients by considering them holistically; 3) minimizing harms to individual patients related to time spent on the waiting list; and 4) consistency within teams, as a way for clinicians to enact fairness within their sphere of agency. Again, the clinicians we spoke with did not always explicitly articulate these “values in action” in the terms presented here. Rather, the clinical ethics team identified ethical values, and applied ethics language to concerns, ideas, and practices expressed by the participants. Below we set out more detailed descriptions of these values in action, as well as considerations for enacting these in practice, that emerged from our conversations with clinicians. These values in action are not novel, but rather, will be recognized by many as a part of good surgical practice. This is an example of applied or practical ethics work that seeks to identify and *clarify* values that are already commonly accepted by relevant stakeholders (Thompson et al. 2006), in order to support ethical practice.

### Supporting Autonomy Through Informed Consent

A common ethical concern expressed by clinicians we spoke with was whether a patient’s decision to pursue surgery was genuinely autonomous. In particular, clinicians spoke about the limitations of informed consent; it can be difficult both to predict and to communicate to a patient how a surgery will impact their life in the short and long-term. They also noted that family members or friends could influence patients’ decision-making around surgery, as could surgeons themselves. A number of clinicians were cognizant that the way they frame risks and potential benefits of surgeries could have a significant impact on patient decision-making. For instance, telling patients that a surgery entails a 10 per cent risk of morbidity tends to be less palatable than telling patients surgery has a 90 per cent chance of being uncomplicated. Another significant ethical concern was that patients on the waiting list who may never receive their surgery could be unaware this is the case, highlighting the importance of transparency.

These concerns revealed, and were countered by, a value in action: supporting autonomy through informed consent. Clinicians were expressing the view that patients should be provided with an estimate of realistic wait times for surgery and support to understand the impact of the proposed surgery on their life, as well as standard information about potential risks, benefits, and alternatives to surgery.

From a clinical ethics perspective, practical considerations and suggestions for enacting this value included:

- Ensure the patient has been given the opportunity, and made to feel comfortable, to ask all the questions they have.
- Offer best possible estimate of waiting time (along with reasonable updates if/when patient is on the waiting list).
- Explore whether accessing surgery through the private system is an option available to the patient to avoid long wait times.
- Acknowledge the difficulty of predicting and communicating the impact of surgery on individuals. Share range of possible outcomes and other patient stories, as appropriate.
- Consider how information, including risks and possible benefits, is presented, noting “framing effects” (equivalent information can be more or less palatable, depending on where emphasis is placed).

### Maximizing Benefits to Individual Patients by Considering Them Holistically

Another notable ethical concern shared by clinicians was reflecting on uncertainty about whether a proposed surgery would improve a patient’s overall quality of life. For instance, a surgery may be technically successful for a patient, and improve survival, but they may not be able to return to living at home independently. This may be an acceptable outcome to some patients but not to others.

The value in action embedded in this concern is maximizing benefits to individual patients by considering them holistically. Clinicians expressed that it was important to consider what a successful outcome means for the individual patient, and whether this will likely be achieved with the proposed surgery. It was also noted that the impact of the whole surgical journey should be taken into account, including time spent on the waiting list.

There are various ways in which this value could be enacted in practice, including:

- Consider potential benefits and risks of surgery broadly, in context of the patient’s life and goals, in conversation with the patient.
  - ⚬ What are the patient’s goals, and can they be achieved with surgery? How likely is it that surgery will achieve the patient's goals?
  - ⚬ How will anticipated time on the waiting list impact the patient’s life?
  - ⚬ Will predicted benefits of surgery improve the patient’s overall quality of life?
- Consider whether there are less invasive alternatives to surgery that might achieve some of the patient’s goals.
- Consider whether a patient’s psychosocial needs should be accounted for in determining urgency of surgery. (Note that whether and how such factors are taken into account should be consistent within teams, as discussed below.)

It is possible that nuanced conversations with individuals about the subjective risks and benefits of surgery for them, and alternative options available, *may* ultimately lead to fewer patients being waitlisted.

### Minimizing Harms to Individual Patients Related to Time Spent on the Waiting List

Clinicians we spoke with expressed a variety of ethical concerns related to the time patients were spending waiting for surgery. It was noted that phone calls to outpatient clinics were sometimes unanswered, leading to inadequate triage and patient communication at this early stage of the patient journey. Another issue of particular concern was that, while on the waiting list, patients’ need for surgery can become more urgent without the team’s knowledge. Clinicians also shared cases of patients’ conditions deteriorating while waiting for surgery, resulting in a more complex intervention being necessary. It was noted that all of these concerns—micro-level responses to which are largely related to patient communication—were heightened for patients with low health literacy.

In an ideal world, many of these concerns would be addressed by increasing resources and reducing patient waiting time. And in discussion of these issues, clinicians did refer to the macro ethical challenge of resource allocation to maximize benefits at a population level, while maintaining equity. However, more attention was focused on actions that could be taken within the constrained system to minimize harms to individuals related to the inevitable time spent awaiting surgery, revealing another value in action.

Some strategies suggested by clinicians for enacting this value included:

- Review and streamline outpatient triage processes.
- Implement calls at regular intervals to patients on the waiting list to review their condition.
- Use patient-reported outcome measures at regular intervals during waiting period to identify patient deterioration.
- Identify a primary contact at the hospital for each patient.
- Consider additional supports for patients with low health literacy that are particularly vulnerable to communication challenges while on the waiting list.

### Consistency Within Teams, as a Way for Clinicians to Enact Fairness Within Their Sphere of Agency

Clinicians expressed a number of ethical concerns related to inconsistency in decision-making, in particular in relation to variability in use of prioritization categories. For instance, some noted that trainees are more likely to assess a patient’s condition as more urgent and use a higher priority category than a more experienced colleague. Some clinicians would take social circumstances into account when assessing urgency and others not. It was also noted that it was common practice for clinicians to informally use subcategories, to indicate that a particular patient should be prioritized or de-prioritized within a category, for instance allocating a patient as category 2(a) or 2(b). These subcategories, which do not form part of the official categorization system in the Victorian Planned Surgery Policy, were noted to be used inconsistently between and within teams. Another concern raised was that the “treat in turn” principle, outlined above, was bypassed in practice for various reasons, often legitimate but requiring scrutiny and consistency. For instance, this principle was balanced against the goal of maximizing theatre usage and meeting the training needs of junior clinicians.

Striving for consistency in decision-making within teams, through transparency and consensus-building, was highlighted as another value in action, allowing clinicians to enact fairness within their sphere of agency.

Strategies for enacting this value require ethical action specifically from team leaders:

- Identify key decisions that require subjective judgments from individuals and facilitate team deliberation on relevant issues. (For example, ongoing discussion and education within teams to ensure consistent understanding and use of urgency categories and subcategories, including relevance of psychosocial factors to categorization.)
- Consider team-specific decision-making frameworks or guidance for regularly encountered prioritization challenges. (For example, balancing optimization of theatre usage with the treat in turn principle).

## An Overall Framework of Values in Action in the Management of Planned Surgery Waiting Lists

**Figure 1:**
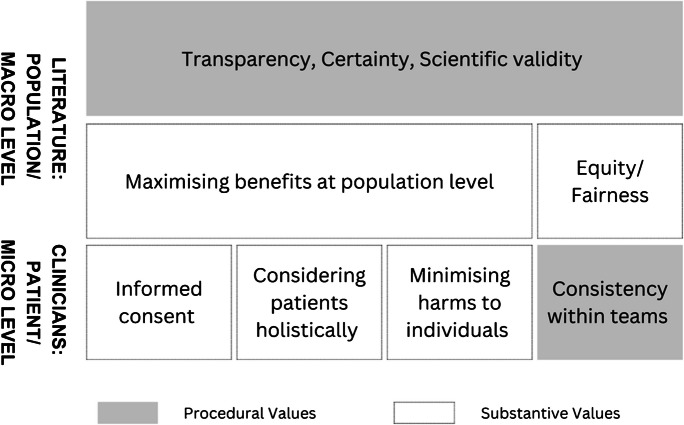
An overall framework of values in action

In Figure 1 we depict a framework of all of the values we identified as at play in the planned surgery context. The framework includes the values gleaned from the literature review, which are geared toward the population or macro level, as well as those identified in conversation with clinicians, which are focused at the patient or micro level. As noted above, the key values that emerged from the literature were broadly accepted by clinicians, however clinicians were more focused on values that were relevant to their sphere of practice and agency. Notably, these micro level values directly served the macro level values. That is, by supporting individual patients in various ways (through informed consent, holistic consideration, and minimizing individual harms) clinicians can contribute to the broader goal of maximizing benefits at a population level from their unique position. Similarly, consistency within teams is a way to enact fairness within their sphere of agency, supporting the broader goal of equity at a population level. Transparency has a role, not only as procedural value requiring prioritization processes to be well-defined and open to scrutiny, but also as a feature of informed consent processes with individual patients.

Decision-making about ethical issues is inevitable in the planned surgery space, and there is no straightforward answer to the ethical challenges that arise in practice. Our intention is that this framework of values, both procedural and substantive, along with the strategies for enacting them identified above, can broadly support clinician decision-making in the planned surgery space by encouraging reflection and discussion about relevant values when there is a challenging decision or ethical concern at hand (Thompson et al. 2006).

## Conclusion

In this paper we present a framework of values to inform ethical decision-making in planned surgery that expands the focus of ethical discussion of this context to include decision-making at the individual clinician level. This framework was derived from both a literature review and iterative, ethicist-led discussions with key surgical staff members in one hospital network in metropolitan Melbourne, Australia. It has been argued that “an ethical framework … is robust to the extent that it reflects the values and beliefs of the decision-makers who refer to it and the values and beliefs of those affected by the decisions being taken” (Thompson et al. 2006, 9). Notably, this framework is a result of collaboration between ethicists and a group of clinicians within one hospital network; it cannot be assumed to reflect values commonly accepted by all decision-makers in planned surgery. However, the clear correlation between micro level clinician values reflected here and broadly accepted population level values articulated in the literature does point towards potential generalizability. It must also be noted that the scope of this project did not extend to engaging patients affected by planned surgery decisions. Seeking feedback from patient stakeholders is an important next step in further developing this framework. That said, the strength of this framework is a) in the ground-up, practice-based process taken to its development, such that it reflects values already accepted by decision-makers in our context; and b) in its unique inclusion of decision-making at the clinician level. We offer this framework for others to engage with, refine with input from the relevant stakeholders in their context, and in this way to support ethical practice among staff working within the constrained and imperfect conditions of the planned surgery space.

## Supplementary Information

Below is the link to the electronic supplementary material.

Supplementary file1 (DOCX 23 KB)

## Data Availability

If required, authors can send relevant documentation to verify the validity of presented data.

## Footnotes

1 As of May 2025, 60, 658 on the waiting list with 82 per cent treated within the recommended timeframe. See https://vahi.vic.gov.au/reports/victorian-health-services-performance/planned-surgery for regularly updated information.
