# Supplementary material for: Ethical Management of Elective Surgery Waiting Lists: A Framework of Clinicians’ Values in Action
Source: J Bioeth Inq. 2025 Oct 13;23(2):261–70. doi: 10.1007/s11673-025-10481-0 (PMC13388331; doi:10.1007/s11673-025-10481-0)
Supplement: Supplementary file 1 — Supplementary file1 (DOCX 23 KB) [file 11673_2025_10481_MOESM1_ESM.docx]

**Method details for critical interpretive review**

An initial search was conducted in March 2023, limited to literature published from 2020 onwards to capture post COVID-19 pandemic literature. In this initial search, titles, abstracts, and keywords were searched in Scopus and PubMed to identify literature that proposes ethical frameworks, principles, or values relevant to the management of planned surgery waitlists. The search terms used in Scopus were (ethic* OR moral* OR bioethic*) AND (“waiting list”) AND (surgery OR surgical OR surgeon*). Similar terms were used in PubMed and duplicate results were removed. A Google Scholar search was also conducted at this time, with the first four pages of hits reviewed. In September 2023, a search strategy for MEDLINE (Ovid) was developed by an experienced librarian. This search was guided by the same research question but was not limited by date. The key search terms used were (ethic* OR moral* OR bioethic*) AND (waitlist* or wait list* or wait* time* or waiting list* or referral* or time factor* or patient select* or priorit* or allocat) AND (Elective Surgical Procedures/).

The inclusion criteria were:

- The publication explicitly identifies ethical challenges, frameworks, principles, or values relevant to the management of elective/planned surgery waitlists.
- The publication is in English.

Publications were excluded if they discussed the management of planned surgery waitlists, but not from an ethical perspective. For instance, a proposed process for prioritising patients on planned surgery waiting lists with the goal of minimising overall waiting time did not meet inclusion criteria. Publication titles and abstracts were screened against inclusion criteria, followed by a full text screen.

Reference lists of the included literature were also screened for relevant titles. If relevant, the full text of the publication was screened against inclusion and exclusion criteria. Recent publications (not yet indexed) of specific bioethics journals were also searched during the period of literature review.

A total of 11 publications were included in this review. Of the included publications, 2 were published in ethics journal and the remainder in clinical journals.

Table. Publications included in the review

| **Publication title** | **Author** | **Year** | **Journal title** |
| --- | --- | --- | --- |
| An international multicenter study of protocols for liver transplantation during a pandemic: A case for quadripartite equipoise. | Chew, Iyer, Kow, Muiesan, Mirza, and Bonney. | 2020 | Journal of Hepatology |
| Priorities, actions and risks in the COVID-19 pandemic: a flash SoMe survey among surgical oncologists. | Cortés-Guiral, Sgarbura, Alyami, Yoshida, Doki, Ishigami, Grass, and Hübner. | 2021 | Pleura and Peritoneum |
| Waiting lists and elective surgery: ordering the queue | Curtis, Russell, Stoelwinder, and McNeil | 2010 | The Medical Journal of Australia |
| The ethics of waiting lists for TAVR procedures | Karamanou, Vrachatis, and Tousoulis. | 2020 | European Heart Journal |
| Surgery during COVID-19 crisis conditions: can we protect our ethical integrity against the odds? | Macleod, Mezher, and Hasan. | 2020 | Journal of Medical Ethics |
| Patients waiting lists and the COVID-19 pandemic: A moral dilemma | Miziara and Galego. | 2021 | Perioperative Care and Operating Room Management |
| Waiting lists: management, legalities and ethics | Pitt, Noseworthy, Guilbert, and Williams. | 2003 | Canadian Journal of Surgery |
| Tackling the COVID elective surgical backlog: prioritising need, benefit or equality? | Pugh, Seah, Carr, and Savulescu. | 2023 | Clinical Ethics |
| Patient prioritisation methods to shorten waiting times for elective surgery: A systematic review of how to improve access to surgery | Rathnayake, Clarke, and Jayasinghe. | 2021 | PLOS One |
| Returning to Elective Orthopedic Surgery During the COVID-19 Pandemic: A Multidisciplinary and Pragmatic Strategy for Initial Patient Selection | Vles, Ghijselings, De Ryck, Meyfroidt, Sweeney, Oosterlinck, Casteels, and Moke. | 2020 | Journal of Patient Safety |
| Revisiting the concept of urgency in surgical prioritization and addressing backlogs in elective surgery provision | Wiebe, Kelley, and Kirsch. | 2022 | Canadian Medical Association Journal |
